# Risk factors of obstructive sleep apnea syndrome in children

**DOI:** 10.1186/s40463-020-0404-1

**Published:** 2020-03-04

**Authors:** Zhifei Xu, Yunxiao Wu, Jun Tai, Guoshuang Feng, Wentong Ge, Li Zheng, Zhe Zhou, Xin Ni

**Affiliations:** 1 Department of Respiratory Medicine, Beijing Children’s Hospital, Capital Medical University, National Center for Children’s Health, Beijing, China; 2 Beijing Key Laboratory of Pediatric Otolaryngology, Head & Neck Surgery, Beijing Children’s Hospital, Capital Medical University, National Center for Children’s Health, Beijing, China; 3 Department of Otorhinolaryngology head and neck surgery, Beijing Children’s Hospital, Capital Medical University, National Center for Children’s Health, 56 Nanlishi Road, Xicheng, Beijing, China; 4 Research Center for Big Data and Engineering, Beijing Children’s Hospital, Capital Medical University, National Center for Children’s Health, Beijing, China

**Keywords:** Obstructive sleep apnea, Risk factor, Obesity, Breastfeeding, Adenotonsillar hypertrophy, Child

## Abstract

**Background:**

The known risk factors of childhood OSAS include tonsillar and adenoidhypertrophy, obesity, craniofacial anomalies, neuromuscular disorders and African-American (AA) ancestry. Whether other factors such as allergic rhinitis (AR), premature, environmental tobacco smoking (ETS) are associated with OSAS are inconsistent in different studies. Our study enrolled children of a broad age range and included potential risk factors of OSAS derived from previous studies and our own experience. Our objective is to identify risk factors of OSAS in children in a clinical setting.

**Methods:**

Children between 2 and 15 years of age exhibiting snoring symptoms who visited the sleep center for polysomnography (PSG) were enrolled. All children completed a questionnaire, physical examination and PSG. The questionnaire included demographic data and information related to potential risk factors for sleep disorders. A physical examination included measurements of height, weight, neck circumference, waist and hip ratio, visual evaluation of the tonsils and the degree of adenoid obstruction. Children with obstructive apnea-hypopnea index (OAHI) ≥ 1 were defined as OSAS.

**Results:**

A total of 1578 children were enrolled and1009 children exhibited OSAS. Univariate analyses showed that snoring occurring for ≥ 3 months, male gender, preterm birth, breastfeeding, obesity, neck circumference ≥ 30 cm, waist/hip ratio ≥ 0.95, tonsillar hypertrophy, and adenoid hypertrophy were associated with OSAS. The proportion of low educational level was higher in parents who breastfed their babies than those who didn’t. Multivariate analysis showed that snoring for ≥ 3 months, male gender, obesity, breastfeeding, tonsillar hypertrophy, and adenoid hypertrophy were associated with OSAS. Confounders such as socioeconomic status, parental occupation, and health-related behaviors should be explored further to investigate the relationship between breastfeeding and OSAS.

**Conclusion:**

The independent risk factors for OSAS in children included snoring ≥ 3 months, male gender, obesity, breastfeeding, tonsillar and adenoid hypertrophy.

The study was registered on Clinical Trials government (NCT02447614). The name of the trial is “Follow-up Studies of Primary Snoring (PS) and Obstructive Sleep Apnea Hypopnea Syndrome (OSAHS) in Chinese Children” and the URL is https://clinicaltrials.gov/.

## Background

Obstructive sleep apnea syndrome (OSAS) is a common condition in children. If left untreated, OSAS can result in neurobehavioral and cardiovascular complications and growth impairment [1]. To develop better strategies for screening and managing OSAS, it is important to identify risk factors for OSAS. Tonsillar and adenoid hypertrophy, male gender, obesity and habitual snoring are considered important factors in the development of OSAS [2–8]. There are also studies indicating OSAS has been associated with several controversial risk factors, such as allergic rhinitis (AR), premature birth, parental smoking, low socioeconomic status and African-American (AA) ancestry [3–5, 9–16]. Moreover, several studies hold that breastfeeding may provide long-term protection against the severity of childhood sleep-disordered breathing [5, 17, 18]. However, numerous other studies exist that do not support these findings. Such discrepancies might be due to race and age differences among various study populations and varying definitions of OSAS. In addition, due to the number of subjects evaluated in majority of these studies was small and the majority of these studies had relatively narrow age ranges, they failed to systematically assessed a wide range of risk factors that was associated with risk of OSAS. For example, OSAS risk factors for a community-based population might differ from those for a hospital-based population [16]. The objective of our study was to identify independent risk factors of OSAS in children in a clinical setting. Our study enrolled children of a broad range of ages and included evaluation of potential OSAS risk factors derived from previous published studies and from our own experience. Consequently, we hypothesized that OSAS is associated with male gender, premature birth, obesity, AR, and adenoid and tonsil size and breastfeeding is a protective factor.

## Methods

Children between 2 and 15 years of age with snoring who underwent polysomnography (PSG) in our center between March 1st, 2016 and February 28th, 2017 were recruited. Exclusion criteria included a history of adenotonsillectomy, craniofacial abnormalities, neuromuscular disease and comorbidities such as Down syndrome, Crouzon syndrome, and Pierre Robin sequence.

### Subjects

The study was performed in Beijing Children’s Hospital. A questionnaire to collect demographic data and information related to suspected risk factors for sleep disorders was designed based on literature review [2–15, 17–19] and our experience. Face-to-face interviews were conducted by research assistants who surveyed parents and completed the questionnaire.

Preterm children were defined as< 36 weeks’ gestational age at birth. AR or asthma was considered positive if the parents reported that the child had this condition diagnosed by a physician. Environmental tobacco exposure smoking (ETS) was considered positive if any family member(s) living in the same place smoked one or more cigarette per day. Family history of snoring was considered positive if parent reported that the child’s parent or sibling snore. Breast feeding was defined if the child was fully breastfed for more than 3 months since birth. A parent with a middle school degree or less formal education was scored as having a low educational level.

### Measurements

A physical examination including measurements of height, weight, neck circumference, waist and hip ratio, and visual evaluation of the tonsils was completed during the evening prior to PSG testing. The waist was measured at mid point between iliac crest and lower end of rib cage at mid-axillary line and the neck at the cricothyroid membrane. Neck and height ratio (NHR) were calculated. A fibro-laryngoscopic examination of the adenoids was performed by an ENT specialist in the outpatient clinic prior to the sleep study. Tonsil size is typically evaluated using the 0–4+ scale described by Brodsky et al. [20], with tonsillar hypertrophy being defined as grades 3+ and 4+ [21]. The degree of adenoid obstruction was documented as a percent obstruction of the measured distance between the anterior and posterior surfaces of the nasopharynx [22]. Body mass index (BMI) was adjusted for age and gender and a BMI > 95^th^percentile was considered obese.

### Polysomnography (PSG)

PSG examinations were performed in the sleep center for all subjects using a Compumedics E-series PSG System (Compumedics, Australia) or Alice 5Diagnostic Sleep System (Respironics, USA). During monitoring, parents were present in the same room as their child during testing and each subject was monitored for at least 7 h. The following tests were conducted: four-channel electroencephalography (EEG) with bilateral central and occipital leads; electrooculography (EOG) to measure eye movements; electromyography (EMG) with submental electrodes; electrocardiography; airflow measurement through the nose via a nasal pressure cannula connected to a pressure transducer; and thoracic and abdominal inductive plethysmography to measure respiratory effort. Oxygen saturation was measured by pulse oximetry via a finger probe (Nellcor, Inc., Hayward, CA or Masimo Co., CA, USA).

PSG was interpreted by two technicians and one pediatrician trained in sleep medicine who were unaware of the clinical findings. Sleep stages were assigned on the basis of the criteria by the American Academy of Sleep Medicine manual [23]. Subjects with obstructive apnea-hypopnea index (OAHI) ≥ 1 were defined as OSAS [24].

### Statistical analysis

Continuous data were converted into categorical data based on their clinical significance; e.g. age was classified into < 6 years and ≥ 6 years and a history of snoring was classified into < 3 months and ≥ 3 months. The neck circumference, neck to height ratio and waist to hip ratio were grouped using cluster analysis. The neck circumference was grouped into < 30 cm and ≥ 30 cm, neck to height ratio was grouped into NHR ≥ 0.25 and < 0.25, and waist to hip ratio was divided into three groups, < 0.9, 0.9–0.95, and ≥ 0.95. Adenoid size was classified into four groups based on the percentage of obstruction of the nasopharygeal airway; e.g. not enlarged,< 50%, 51–75%, and ≥ 75%. All categorical data were expressed as frequencies and percentages. The univariate analysis of categorical data was compared using a χ^2^ test and all risk factors with *P* < 0.1 were included in multiple analyses conducted using logistic regression. All statistically significant factors were reported as ORs (odds ratios) with 95%CIs (confidence intervals). A two-sided *P* value of less than 0.05 was considered statistically significant and all analyses were performed using SAS version 9.4.

## Results

A total of 1583 children met the inclusion criteria; however, five of these children were excluded due to either missing data in their questionnaires or due to the lack of PSG results. Of the 1578 subjects who were included in the study, 1008 (63.9%) children exhibited OSAS and 570 (36.1%) were non-OSAS. The average age of OSAS children was 5.7 years old and 701 (69.5%) were boys. The average age of non-OSAS children was 6.0 years old and 359 (62.9%) were boys. There were 324 (32.1%) obese children in OSAS group and 116 (20.4) in non-OSAS group. Table 1 illustrates the demographic data of the subjects.

**Table 1:** Demographic and PSG data of OSAS and non-OSAS children

	OSAS (*n* = 1008)	Non-OSAS (*n* = 570)	*P* value
Age (years)	5.7 ± 2.3	6.0 ± 2.5	0.04
Gender n(%)	701 (69.5%)	352 (61.8%)	< 0.01
BMI (kg/m^2^)	17.8 ± 5.5	16.8 ± 3.1	< 0.01
Obese n(%)	324 (32.1%)	116 (20.4%)	< 0.01
SE (%)	85.6 ± 8.9	86.1 ± 9.1	0.3
OAI (/h)	5.4 ± 10.5	0.1 ± 0.3	< 0.01
OAHI (/h)	10.5 ± 15.3	0.4 ± 0.3	< 0.01
CAI (/h)	1.2 ± 2.0	0.6 ± 0.7	< 0.01
TArI(/h)	5.6 ± 5.5	3.4 ± 3.8	< 0.01
Lowest SaO_2_ (%)	85.8 ± 11.4	92.1 ± 8.0	< 0.01

*BMI* body mass index, *SE* sleep efficiency, *OAI* obstructive apnea index, *OAHI* obstructive apnea-hypopnea index, *CAI* central apnea index, *TArI* total arousal index

The potential risk factors for OSAS are described in Table 2. Univariate analyses showed that snoring occurring for ≥ 3 months, male gender, preterm birth, breastfeeding, obesity, neck circumference ≥ 30 cm, neck/height ratio (NHR) ≥ 0.25, waist/hip ratio ≥ 0.95, tonsillar hypertrophy and adenoid hypertrophy were associated with OSAS. In contrast, family history of snoring, parental educational level, ETS, AR, etc. were not associated with OSAS. In addition, compared with non-breastfeeding parents, breastfeeding parents had a higher proportion of low educational level; The proportion of mothers with low educational level reported breastfeeding their babies versus non-breastfeeding was 41.65% vs. 30.12% (*P* < 0.0001) respectively. The proportion of fathers with low educational level reported breastfeeding their babies versus non-breastfeeding was 41.85% vs. 32.12% (P < 0.0001) respectively.

**Table 2:** Univariate analysis of potential risk factors for OSAS in children

Risk factors	OSAS	*Non-* OSAS	Chi-square	*P* value
Snoring ≥3 months	12.7%	7.9%	8.792	0.0030
Age < 6 years	63.3%	58.8%	3.149	0.0760
Male gender	29.8%	37.7%	10.369	0.0013
Preterm birth	7.5%	4.9%	4.074	0.0435
Breastfeeding	21.4%	18.2%	12.355	0.0021
Family history of snoring	75.8%	75.1%	0.074	0.7859
Low father’s educational level	18.8%	17.5%	2.165	0.3387
Low mother’s educational level	18.4%	16.1%	1.828	0.4009
ETS	53.6%	48.8%	3.555	0.0594
AR	15.7%	15.1%	0.358	0.8359
Obesity	69.5%	20.4%	25.431	<.0001
Neck circumference ≥ 30 cm	14.0%	9.5%	6.826	0.0090
Neck /height ratio ≥ 0.25	23.3%	5.7%	22.600	<.0001
Waist/hip ratio ≥ 0.95	27.5%	21.9%	10.345	0.0057
Tonsillar hypertrophy	79.3%	57.2%	112.815	<.0001
Adenoid hypertrophy	66.0%	58.8%	22.120	<.0001

*ETS* environmental tobacco smoking, *AR* allergic rhinitis

Multivariate analysis showed that snoring for ≥ 3 months, male gender, breastfeeding, obesity, tonsillar hypertrophy and adenoid hypertrophy were associated with OSAS. Table 3 summarizes the results of logistic regression analyses after including age, gender, and significant covariates from the unadjusted models.

**Table 3:** Multivariate analysis of risk factors for OSAS in children

Risk factors	OR	*P* value	lower limit of 95%	upper limit of 95%
Snoring ≥3 months	1.4768	0.0497	1.0005	2.1798
Age <6 years	1.0769	0.0940	0.9472	2.0113
Male gender	1.2715	0.0470	1.0032	1.6117
Preterm	1.5934	0.0567	0.9868	2.5731
Breastfeeding	1.7213	0.0007	1.2563	2.3584
ETS	1.3151	0.1054	0.9440	1.8321
Obesity	1.7062	0.0003	1.2734	2.2862
Neck circumference ≥ 30 cm	1.0195	0.9272	0.6741	1.5416
Neck /height ratio ≥ 0.25	0.7687	0.1016	0.5587	1.0529
Waist/hip ratio ≥ 0.95	1.2053	0.2132	0.8983	1.6173
Tonsillar hypertrophy	4.1552	<.0001	3.0671	5.6292
Adenoid hypertrophy	1.4092	0.0189	1.0582	1.8766

## Discussion

We recruited children between 2 and 15 years of age with snoring to our sleep center during 1 year of period. Because all of these children enrolled for sleep study had symptoms or signs of upper airway obstruction and most of them were referred by ENT or respiratory specialist, the proportion of OSAS was high in this hospital-based population. Our current study found that the independent risk factors for OSAS in children included snoring for ≥ 3 months, male gender, breastfeeding, obesity, tonsillar hypertrophy and adenoid hypertrophy. The unique contribution of this study is its assessment of a wide range of potential risk factors for children across a large age range in a pediatric clinical setting.

Although no consensus exists regarding the optimal duration of a sleep study investigating snoring, this study showed that snoring for ≥ 3 months was predictive of OSAS. Researchers agree that snoring is common in children and that occasional snoring that accompanies an upper respiratory tract infection is of low concern [1]. In line with these findings, some children in this study snored after a viral infection (e.g., EBV) but their symptoms improved in one or 2 months. Therefore, in light of limited medical resources, we suggest that children who snore for longer than 3 months need to see a sleep professional and may need a sleep study.

Gender is another possible risk factor for childhood OSAS, since studies in adults have consistently confirmed that OSA is more common in men than women [25, 26]. However, in children some studies have reported a higher incidence of OSA in boys than girls, while others did not [4, 10, 27, 28]. Indeed, male gender was associated with OSAS in our study, but the mechanism underlying this bias is still unclear. Other researchers have postulated that fat distribution, length and collapsibility of the upper airway, neurochemical control mechanisms, and arousal response might all contribute to the disparity in prevalence between genders [26].

Previous study demonstrated that breastfeeding is a protective factor for OSAS. Children who were breast fed for 2 to 5 months exhibited reduced OSA severity than children who had not been breast fed [17]. Moreover, the author speculated that the act of breastfeeding promoted the development of a healthy upper airway structure and that breast milk provided immunologic protection against infections that promote OSAS. Similarly, Beebe et al. found that children who were fed breast milk, especially for longer periods, were at markedly lower risk for persistent snoring, even after controlling for potentially confounding variables [28].

Surprisingly, breastfeeding was positively associated with OSAS in our current study. This result was similar to one previous study which report breastfeeding was a risk factor of habitual snoring [5]. Moreover, further analysis in our study demonstrated that parents of a lower educational level were more likely to breastfeed their children than parents of a higher educational level. Meanwhile, previous studies had reported that low socioeconomic status was a strong predictor of snoring and sleep-disordered breathing (SDB) [14, 15, 28]. The possible reasons underlying this correlation included reduced access to health care, differences in health-related behaviors, and exposure to environmental toxicants. Nevertheless, breastfeeding in this study still remained a significant risk factor after results were adjusted for educational level. We speculated that although parental educational level and socioeconomic status were closely related, they could not replace one another. Interestingly, a previous study had reported that parents with academic occupations and parents who were farmers exhibited decreased risk of SDB in offspring [29]. Confounders such as socioeconomic status, parental occupation, access to health service, inflammation, environmental exposure, and health-related behaviors should therefore be explored further to investigate the relationship between breastfeeding and OSAS.

Obesity is another risk factor for childhood OSAS that has been identified in several studies [24, 30–32]. Our study observed that obesity, defined as BMI above the 95^th^percentile, was significantly related to OSAS in children. Meanwhile, Katz et al. reported that neck to waist ratio, an index of body fat distribution, predicts OSA in older children and youth who are obese or overweight [33]. Similarly, we found neck circumference ≥ 30 cm, neck/height ratio ≥ 0.25 and waist/hip ratio ≥ 0.95 were associated with OSA. However, when neck circumference, neck/height ratio ≥ 0.25 as well as waist/hip ratio were entered into the multiple regression analysis, none of them remained significant. Because the mean ages of our study population were 5.7 yrs. and 6 yrs. for OAS and non-OAS subjects, respectively, we assume that adipose accumulation around the neck and waist might play a lesser role in younger children, who tend to be less obese, than in adults or adolescents.

In contrast, several other studies did not demonstrate an association between obesity and OSAS in children [10, 11, 16]. Rosen and her colleague observed a significant association between obesity levels and OSAS severity in unadjusted analyses; however, the result was not significant in the adjusted models. Weinstock didn’t find an association between obesity and AHI in the overall sample, but a significant positive association between obesity and AHI in the non-AA group was observed in the race-stratified analyses [16]. These exploratory analyses suggest that risk factors for OSAS may differ across population groups.

Numerous studies have investigated the relationship between adenotonsillar hypertrophy (ATH) and OSA [6, 7, 34–36], ATH is currently considered the most important risk factor for developing OSA in children. Hypertrophy of the tonsil and/or adenoid results in upper airway narrowing and, when superimposed with other factors (e.g., reduced muscle tone), can lead to a clinically significant dynamic airway obstruction during sleep [1]. Our study was in agreement with these previous studies showing that adenoid and tonsillar hypertrophy are associated with OSA in children.

Preterm birth has also been investigated as an OSA risk factor. Rosen et al. observed that SDB occurred with an almost 3-fold greater frequency in 8- to 11-year-old children born preterm compared with children born full term [7]. Similarly, Tapia et al. reported an increased prevalence of OSAS in school-aged children who were born preterm [11]. Moreover, former preterm children may be at increased risk for SDB partly based on their exposures in the perinatal environment; such exposures may influence the development of respiratory control or upper airway size [7]. In this study we found a positive association between premature birth and OSAS using univariate analysis, but this association was not significant after multivariate analysis. This was most likely because that our sample was clinically based and the number of preterm children was small (7% in OSAS and 5% in controls). Therefore, the role of premature birth in the general population needs further investigation.

Allergic rhinitis was reported as a risk factor of OSAS since it is associated with at least partial nasal passage obstruction. A recent systemic review reported that the majority of studies showed a significant association between AR and SDB [37]. Indeed, chronic sinusitis/rhinitis was shown to be a risk factor for OSAS or snoring in children in the general population [5, 10, 27]. However, the current study did not find an association between AR/sinusitis and OSAS. Similarly, Weinstock et al. did not find an association between AR and OSAS in children who were hospital inpatient candidates for adenotonsillectomy [16]. The presence of enlarged tonsils and adenoids as a dominant factor in the genesis of OSAS might well render the relative contribution of AR to be less and more difficult to identify.

As a final note, this study had limitations. First, facial structure abnormalities such as mid-face hypoplasia and micrognathia were not included in our models. However, such physical deformities could be significant risk factors for OSAS for a specific child. Secondly, information regarding AR/sinusitis and asthma as reported by parents were not verified via thorough medical evaluation of nasal abnormalities and documentation in medical records.

## Conclusion

In this study, the significant risk factors for OSAS in children include snoring ≥ 3 months, male gender, obesity, tonsillar and adenoid hypertrophy and, surprisingly breast feeding. Further studies are warranted to confirm the relationship between breastfeeding and OSAS and the mechanisms behind this relationship.

## Data Availability

The datasets used and analysed during the current study are available from the corresponding author on reasonable request.

## Abbreviations

AA: African-American
AR: Allergic rhinitis
ATH: Adenotonsillar hypertrophy
BMI: Body mass index
EEG: Electroencephalograph
EMG: Electromyograph
EOG: Electrooculograp
ETS: Environmental tobacco smoking
NHR: Neck and height ratio
OAHI: Obstructive apnea-hypopnea index
OSAS: Obstructive sleep apnea syndrome
PSG: Polysomnography
SDB: Sleep disordered breathing
